# Association of gut microbiota with portal vein pressure in patients with liver cirrhosis undergoing living donor liver transplantation

**DOI:** 10.1002/jgh3.13018

**Published:** 2023-12-09

**Authors:** Katsuya Toshida, Shinji Itoh, Yukiko Kosai‐Fujimoto, Takuma Ishikawa, Yuki Nakayama, Yuriko Tsutsui, Norifumi Iseda, Takuma Izumi, Yuki Bekki, Shohei Yoshiya, Takeo Toshima, Makoto Nakamuta, Tomoharu Yoshizumi

**Affiliations:** ^1^ Department of Surgery and Science, Graduate School of Medical Sciences Kyushu University Fukuoka Japan; ^2^ Department of Gastroenterology, Kyushu Medical Center National Hospital Organization Fukuoka Japan

**Keywords:** gut microbiota, living donor liver transplantation, portal vein pressure, propionate

## Abstract

**Background and Aim:**

Many recent studies have shown a relationship between various systemic diseases and the gut microbiota (GM), with the gut–liver axis receiving particular attention. In contrast, no report has comprehensively shown the effects of GM on the pathophysiology of patients undergoing living donor liver transplantation (LDLT).

**Method:**

We enrolled 16 recipients who underwent LDLT for liver cirrhosis, and 17 donors constituted the reference group. We examined the differences in GM between recipients and donors. We also examined the relationships between GM, short‐chain fatty acids, and portal vein pressure (PVP) in recipients.

**Results:**

There was no significant difference in alpha‐diversity between the recipients and donors, but there was variation in beta‐diversity among the recipients. The abundance of the phylum Bacteroidetes was significantly higher in recipients than in donors (*P* = 0.016), and it was positively correlated with PVP (*r* = 0.511, *P* = 0.043). Propionic acid, which is a component of short‐chain fatty acids, was positively correlated with PVP (*r* = 0.544, *P* = 0.0295), the phylum Bacteroidetes (*r* = 0.677, *P* = 0.004), and total bilirubin concentration (*r* = 0.501, *P* = 0.048). Propionic acid was negatively correlated with serum albumin concentration (*r* = −0.482, *P* = 0.043).

**Conclusion:**

Our findings suggest relationships between fecal Bacteroidetes levels, propionic acid concentrations, and PVP in patients with liver cirrhosis undergoing LDLT.

## Graphical abstract

In liver cirrhosis patients who have undergone liver transplantation, the phylum Bacteroidetes in gut microbiota is upregulated. This phylum has a significant correlation with high propionic acid level and high portal vein pressure.

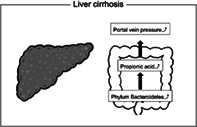

## Introduction

Liver cirrhosis represents a major health burden worldwide, and the most common causes of cirrhosis are hepatitis C, alcohol‐associated liver disease, and non‐alcohol‐associated liver disease.^1^ Liver transplantation (LT) has been accepted as the most effective treatment for end‐stage liver disease,^2^, ^3^ and various studies have shown improvement in the prognosis after LT.^4^

The gut microbiota (GM) contributes to various physiological processes such as nutrient metabolism, maintenance of gut barrier integrity, immune modulation, and production of bioactive metabolites.^5^ Recent advances in research have highlighted the intricate relationship between GM and liver function, emphasizing the importance of the gut–liver axis in the pathogenesis of liver cirrhosis.^6^ Dysbiosis, characterized by alterations in the composition and diversity of the GM, has been observed in patients with liver cirrhosis, suggesting a potential role of GM in disease progression.^7^

Short‐chain fatty acids (SCFAs) are a group of bioactive metabolites produced by GM through the fermentation of dietary fibers and other complex carbohydrates.^8^ In the colon, acetic acid, propionic acid, and butyric acid are present in proportions of 60%, 25%, and 15%, respectively. SCFAs have been implicated in diverse biological functions such as energy homeostasis, modulation of immune responses, and maintenance of intestinal barrier integrity.

To the best of our knowledge, there have been no reports relating GM to portal vein pressure (PVP) in patients undergoing living donor liver transplantation (LDLT). To investigate this relationship, we conducted a prospective observational study on patients undergoing LDLT.

## Materials and methods

### Patients and specimen preparation

This prospective study was approved by the ethics committee of Kyushu University Hospital (approval number: 2019‐067). All procedures were conducted in accordance with the Declaration of Helsinki, 2013. We enrolled recipients who had undergone LDLT for liver cirrhosis at Kyushu University Hospital from June 2017 to December 2018. The reference group consisted of donors who also enrolled in the study in the same period. Data of the patients' factors [age, sex, body mass index, etiology (hepatitis B or C infection, non‐alcoholic steatohepatitis, primary biliary cholangitis], preoperative medications (β‐blockers, rifaximin, antibiotics, proton pump inhibitor, ursodeoxycholic acid), The Model for End‐Stage Liver Disease score, ABO‐incompatibility, hepatocellular carcinoma (HCC), and liver fibrosis markers [hyaluronic acid, type IV collagen, Mac‐2 binding protein glycosylated isomer ***s*** (M2BPGi), fibrosis‐4 index (FIB‐4 index)] were recorded.

### Surgery and postoperative management

The graft harvesting technique, recipient surgery, perioperative management, and immunosuppression regimens have been previously described.^3^, ^9^ Portal vein cannulation for intraoperative PVP monitoring was performed using a 16‐gauge pressure monitoring cannula via one of the terminal jejunal veins immediately after a laparotomy. Immunosuppressive treatment was initiated with mycophenolate mofetil within 24 h after LDLT in all the recipients. Tacrolimus or cyclosporin A was added within 48–96 h. Methylprednisolone 1000 mg was administered after graft reperfusion and was tapered to reach 5 mg of oral prednisone within 1 month. Only patients who underwent ABO‐incompatible LDLT received rituximab 3 weeks before operation. Perioperative prophylaxis consisted of intravenous cefotaxime and cefazoline for 48 h in recipients, and intravenous cefazoline during the surgery. Both these agents were started just before the skin incision was made. In recipients with pre‐LDLT infections, the administration of pre‐LDLT antibiotics was continued during and after LDLT.

### Sample collection and microbiome analysis

**Figure 1:**
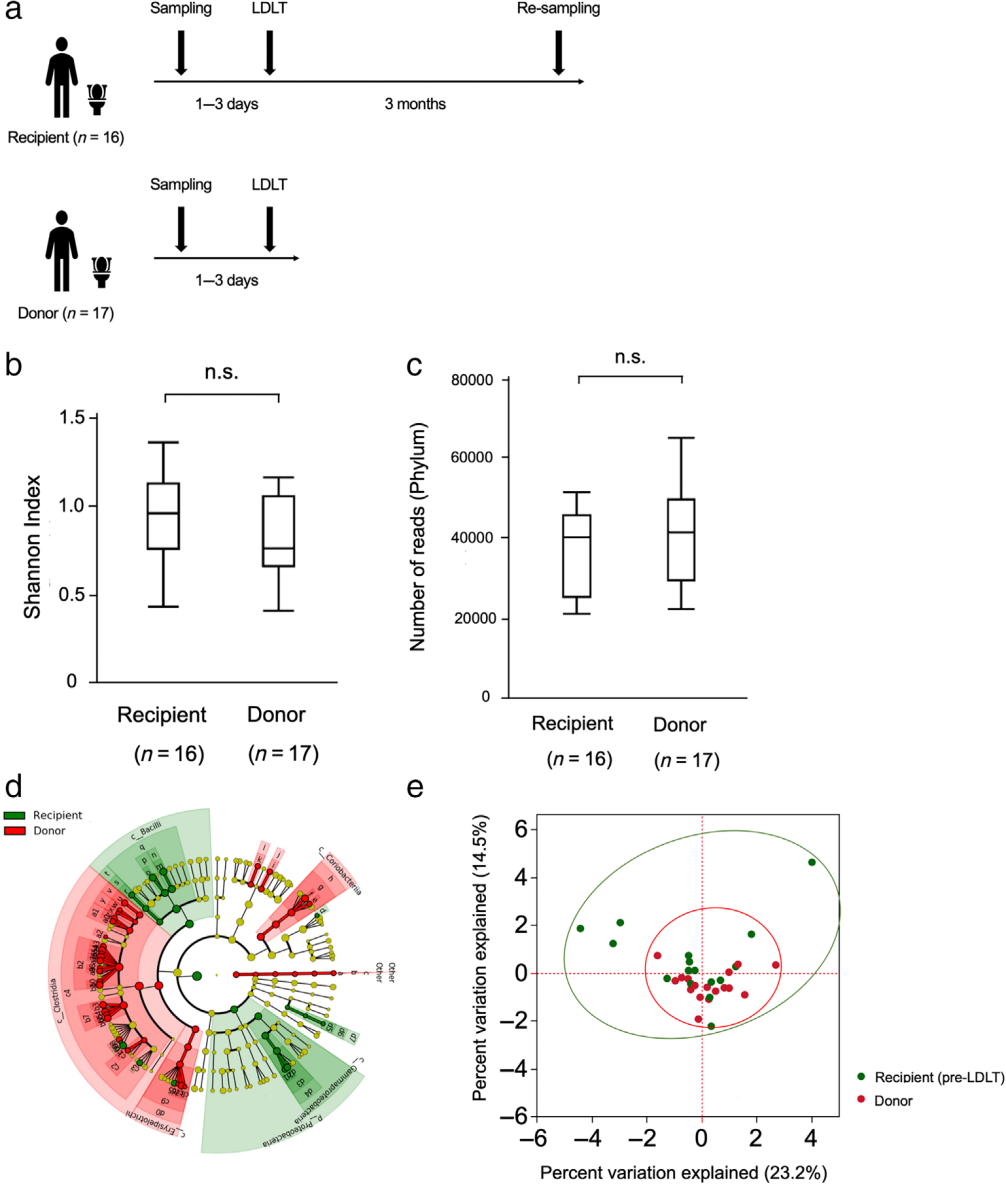
Protocol and diversity of GM. (a) Protocol of collection of fecal samples from recipient and donor. (b) Shannon index of fecal samples between recipient and donor, showing no significant difference. (c) Leads number of fecal samples (phylum) between recipient and donor, showing no significant difference. (d) Cladogram of the recipient and donor GM. (e) Principal coordinate analysis to confirm the beta‐diversity between recipient and donor. GM, gut microbiota.

Fecal samples were collected from the recipients and donors 1–3 days before surgery and postoperatively at 3 months in the recipients using a fecal sample collecting kit (Fig. 1a). The feces from individual patients were kept frozen at −80°C until analysis. Next‐generation 16S ribosomal ribonucleic acid sequencing was then performed. Deoxyribonucleic acid was extracted from the samples using the beads‐phenol method.^10^ Analyses of sequence reads were manually performed using the Ribosomal Database Project Multiclassifier tool, which is available from the RDP website (http://rdp.cme.msu.edu/classifier/).^11^ Reads obtained in the FASTA format were assigned to class levels with an 80% confidence threshold.

### SCFA analysis

SCFA concentrations were analyzed at LSI Medience Corporation (Tokyo, Japan), which is a contract laboratory. Briefly, approximately 150 mg of tissue or feces was transferred to a disruptor tube supplied by Yasui Kikai (Osaka, Japan) and shaken with an iron cone cooled in liquid nitrogen. The powders were suspended with 1 mL of water, and 50 μL of the suspension was transferred to a micro tube (when a specimen was serum, 50 μL of serum was transferred into a micro tube). After mixing with 200 μL of methanol using a shaker for 15 min, the samples were centrifuged at 20,000*g* for 1 min at 4°C. A volume of 200 μL of the supernatant was mixed with 2‐nitrophenylhidrazine and 1‐3‐dimethylaminopropyl‐carbodiimide solution and analyzed by liquid chromatography–tandem mass spectrometry (Nexera X2 LC‐30 AD, 8050; Shimadzu, Kyoto, Japan) equipped with a reverse‐phase LC column (ACQUITY UPLC HSS T3,1.8 μm, 2.1 × 50 mm; Waters, USA). The data were analyzed using the LabSolutions software (Shimadzu). The peak areas were normalized by internal standards, and the concentration of each SCFA was obtained using a standard curve.

## Results

### Characteristics of recipients and donors

The demographic features of the patients are shown in Table 1. This study included 16 recipients and 17 donors (as a control group), with a median recipient age of 55 years (range: 24–68 years) and a median donor age of 42 years (range: 27–62 years). Nine recipients were men and 12 donors and were men. The primary hepatic diseases were viral hepatitis, non‐alcoholic steatohepatitis, alcoholic steatohepatitis, primary biliary cholangitis, and others. Most recipient (87.5%) had preoperative medication of ursodeoxycholic acid. The median MELD (Model for End‐Stage Liver Disease) score was 16 (range: 10–26). Detailed clinical characteristics of each recipient including PVP are shown in Table S1 (Supporting Information).

**Table 1:** Clinical characteristics of recipient and donor

	Recipient (*n* = 16)
Age (years)	55 (24–68)
Sex, male/female	9/7
Body mass index	22.6 (17.3–26.5)
Etiology	
Hepatitis B or C infection	7 (43.8%)
Non‐alcoholic steatohepatitis	2 (12.5%)
Alcoholic steatohepatitis	3 (18.8%)
Primary biliary cholangitis	2 (12.5%)
Others	3 (18.8%)
Preoperative medications	
β‐Blocker	0 (0.0%)
Rifaximin	6 (37.5%)
Antibiotics	2 (12.5%)
Proton pump inhibitor	8 (50.0%)
Ursodeoxycholic acid	14 (87.5%)
MELD score	16 (10–26)
ABO incompatibility	7 (43.8%)
Hepatocellular carcinoma	5 (31.3%)
	Donor (*n* = 17)
Age (years)	42 (27–62)
Sex, male/female	12/5

Data are presented as *n* (%) or median (range).

MELD, Model for End‐Stage Liver Disease.

### Diversity of GM between recipients and donors

Alpha‐diversity was quantified using the Shannon diversity indices, which relate operational taxonomic unit richness and evenness. There was no significant difference in the Shannon index or the number of reads (phylum) between the recipients and donors (Fig 1b,c). Figure 1d shows the differentially abundant GM among recipients and donors. Furthermore, a principal coordinate analysis was performed to determine the beta‐diversity (Fig 1e). This analysis showed that the recipient GM had greater diversity than the donor GM.

### Comparison of GM components between recipients and donors

**Figure 2:**
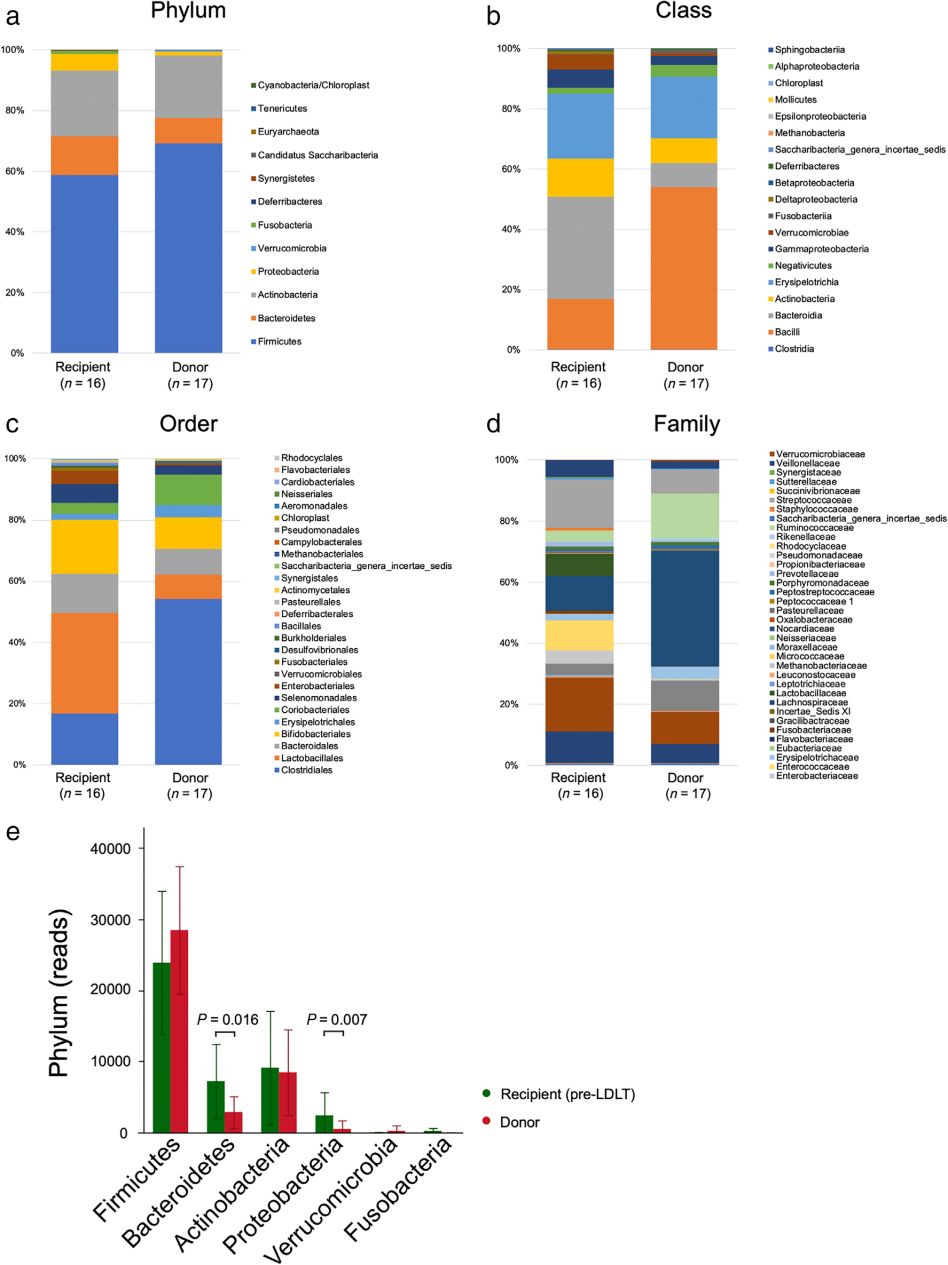
Difference of GM component (phylum/class/order/family). (a) Phylum, (b) class, (c) order, (d) family. (e) Comparison of GM (phylum) between recipient and donor.

The composition of the GM (phylum/class/order/family) is shown in Figure 2a–d, respectively. There was no significant difference in the relative abundance of Firmicutes between recipients and donors. However, the relative abundance of Bacteroidetes and Proteobacteria was significantly higher in recipients than in donors (*P* = 0.016, *P* = 0.007, respectively) (Fig 2e).

### Analysis of GM in subgroups

We performed two subgroup analyses about GM composition for deeper understanding.

Because the existence of tumor may affect the GM, we first analyzed the result of the Shannon index (Fig S2a), the number of reads (Fig S2b), phylum (reads) (Fig S2c) between recipients with HCC and healthy donors. However, there was no significant difference. Next, because alcohol may affect GM, we analyzed the result of Shannon index (Fig S2d), number of reads (Fig S2e), and phylum (reads) (Fig S2f) between recipients whose etiology is alcohol or non‐alcohol. However, there was no significant difference.

### Effect of GM and SCFA on PVP

**Figure 3:**
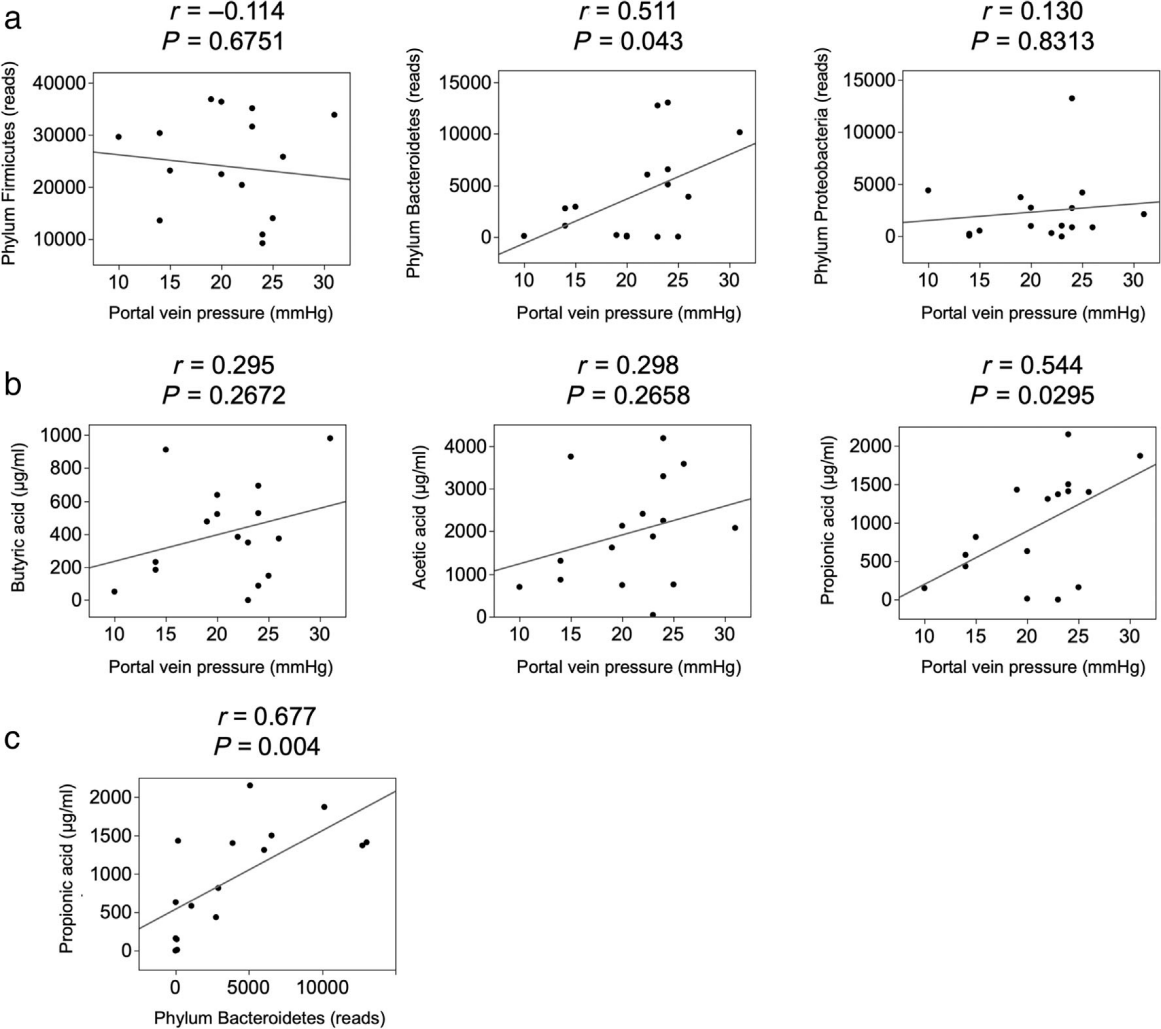
Relationship between GM, SCFA, and PVP. (a) Correlation between GM (phylum) and PVP. (b) Correlation between fecal SCFA and PVP. (c) Correlation between fecal propionic acid and the phylum Bacteroidetes. GM, gut microbiota; PVP, portal vein pressure; SCFA, short‐chain fatty acids.

The composition of SCFAs is shown in Figure S1. Acetic acid, 3‐hydroxybutyric acid, and lactic acid comprised the majority of serous SCFAs. However, acetic acid, propionic acid, and butyric acid were the main fecal SCFAs. We examined the relationship between GM and PVP in recipients. The relationship between the phyla Firmicutes, Bacteroidetes, and Proteobacteria, which are the main components of GM, and PVP was examined. There was a positive correlation between Bacteroidetes and PVP (*r* = 0.511, *P* = 0.043) (Fig 3a). There was no significant correlation between serous SCFAs and PVP, but patients with high PVP tended to have a low level of acetic acid concentration (Fig S3). In the relationship between fecal SCFAs and PVP, a positive correlation was observed between propionic acid and PVP (*r* = 0.544, *P* = 0.0295) (Fig 3b). We also observed a positive correlation between Bacteroidetes and propionic acid in recipients (*r* = 0.677, *P* = 0.004) (Fig 3c). In donors, we did not find a significant correlation between Bacteroidetes and propionic acid (Fig S4). Finally, we examined the relationship between propionic acid and peripheral blood laboratory values reflecting liver function. There was a significant positive correlation between propionic acid and total bilirubin concentrations (*r* = 0.501, *P* = 0.048) and a negative correlation between propionic acid and albumin concentrations (*r* = −0.482, *P* = 0.043) (Fig S5).

### Effect of liver fibrosis markers on PVP and GM

After showing that the composition of GM was associated with portal pressure, we examined whether the serum‐based liver fibrosis markers were associated with PVP and GM. Of the 16 recipients, we could collect the data (hyaluronic acid, type IV collagen, Mac‐2 binding protein glycosylated isomer**s** (M2BPGi), FIB‐4 index) from 14 recipients. Between type IV collagen and PVP, there was positive tendency, but there were no significant correlations between serum‐based liver fibrosis markers and PVP (Fig S6a). Next, we examined the association between serum‐based liver fibrosis markers and the phylum Bacteroidetes, but there were no significant correlations (Fig S6b).

### Change of GM after LDLT

**Figure 4:**
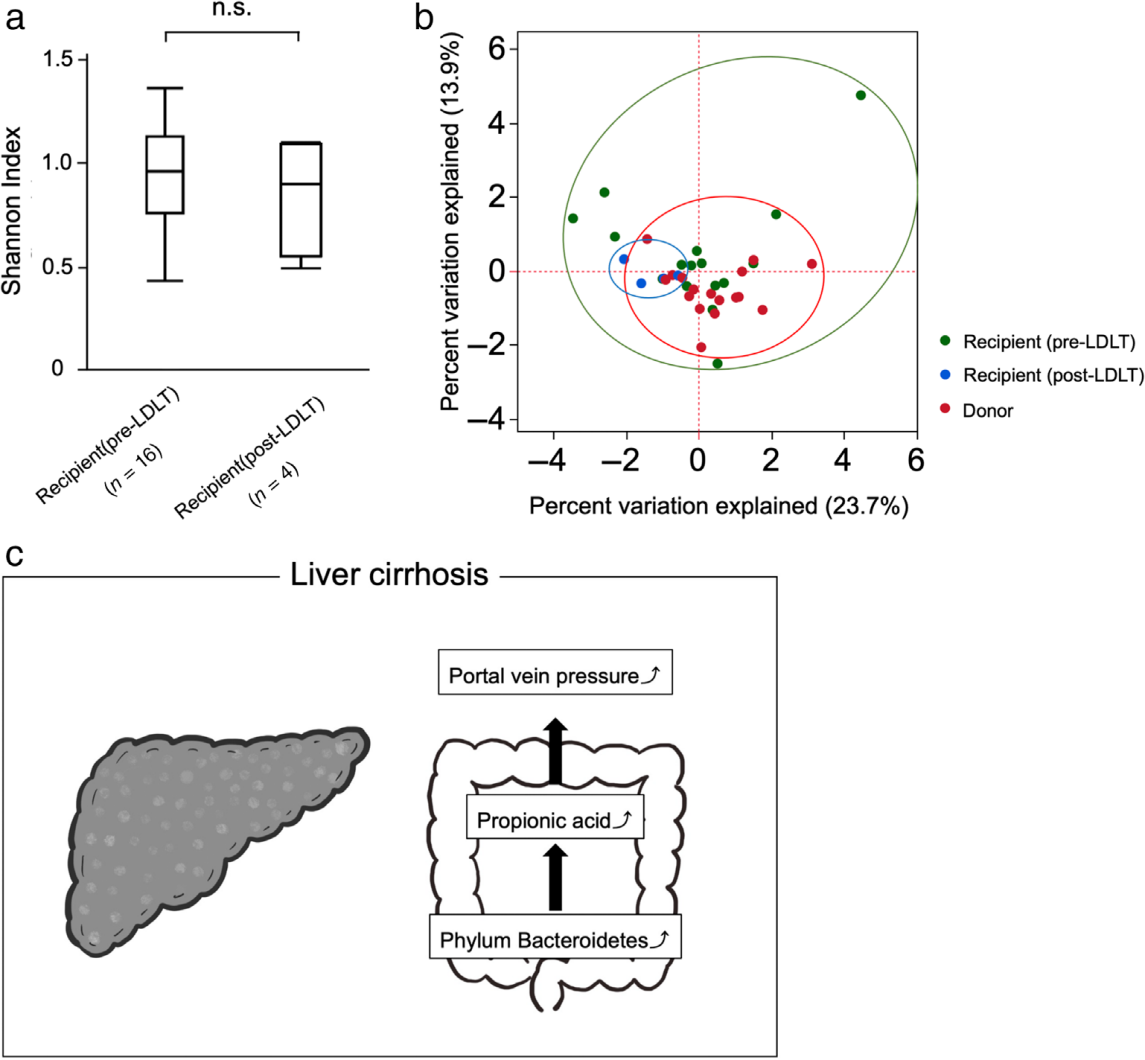
Pre‐ and post‐operative fecal diversity changes before and after LDLT and summary of this paper. (a) Shannon index of fecal samples before and after LDLT, showing no significant difference. (b) Principal coordinate analysis to confirm the beta‐diversity before and after LDLT. (c) Graphical abstract LDLT, living donor liver transplantation.

We collected fecal samples from four patients after LDLT and compared the pre‐ and post‐operative alpha‐ and beta‐diversity. There was no significant difference in the alpha‐diversity between the pre‐ and post‐operative GM (Fig 4a). The patients showed a clustered state of beta‐diversity after operation compared with before operation (Fig 4b).

## Discussion

In the present study, we examined GM and SCFAs in recipients who had undergone LDLT and donors, and showed their effect on PVP. We found that GM in recipients changed after LDLT.

The human microbiome comprises every gene from bacteria, archaea, viruses, and eukaryotic microbes, most of which reside in the gut. The bidirectional association between the gut and liver indicates the involvement of the gastrointestinal microbiome in the progression of chronic liver disease.^12^ The human GM is broadly divided into several phyla, and the most clinically relevant phyla in patients with liver cirrhosis are Firmicutes, Bacteroidetes, and Proteobacteria.^13^ Previous studies have shown that the number of bacteria in Bacteroidetes was associated with the progression of some liver diseases and that there were differences in the number of bacteria in Firmicutes, resulting in an increased Firmicutes/Bacteroidetes ratio.^14^, ^15^ In the present study, no significant changes in the abundance of Firmicutes were observed in GM of the recipients. However, there was a significant increase in the abundance of Bacteroidetes in recipients, which is consistent with previous reports.

The association between portal hypertension and GM has been reported.^16^ The number of cases in this study was small because the measurement of PVP is an invasive procedure for patients, so our finding of the associations between PVP measured by cannulation during the operation and the GM and SCFAs is important. We observed positive correlations between PVP and the abundance of Bacteroidetes and propionic acid. This finding suggests a role of propionic acid, which is a metabolite possibly derived from Bacteroidetes, in regulating portal hypertension. The associations between Bacteroidetes, propionic acid, and portal hypertension may have several explanations. First, Bacteroidetes changes the components of SCFAs by producing SCFA, particularly propionic acid.^17^ SCFAs modulate endothelial function, immune responses, and vascular tone. An imbalance in SCFA production due to dysbiosis could disrupt these regulatory mechanisms, contributing to portal hypertension. Second, a previous study found that intraportal administration of propionic acid increased PVP in animals.^18^ The mechanism by which propionic acid regulates PVP has not been determined, but the results are consistent with clinical practice.

With regard to the control of PVP, we have previously demonstrated the importance of simultaneous splenectomy in small‐for‐size graft syndrome during liver transplantation.^3^, ^19^ Some preoperative interventions to control PVP may improve the prognosis after LT because high PVP leads to not only greater intraoperative blood loss and a longer duration of surgery but also to a worse postoperative prognosis.^20^, ^21^ In our study, the beta‐diversity in fecal samples after LDLT was less variable than before LDLT, and we found that the GM in recipients could be changed by LDLT (Fig 4b). However, a limitation to this study is that only a few patients were able to have fecal samples collected postoperatively. Therefore, a large‐scale study is required to compare the GM before and after LDLT, and to determine how the composition of the GM and SCFAs are altered and affected. Additionally, methods, such as preoperative medications or fecal transplantation, could be useful for changing the GM in recipients. Fecal transplantation has recently been shown to improve sarcopenia,^22^ which may have a positive effect not only on PVP but also systemically, improving the prognosis after LDLT.

There are several limitations to this study. First, the study size was small, and a sub‐analysis by background liver diseases could not be performed. Because GM may vary significantly with each background liver disease, larger prospective studies are needed in the future. The second limitation is that the number of postoperative samples was small. A sufficient size of postoperative samples would provide further information regarding propionate production and GM composition after liver transplantation.

## Conclusion

In conclusion, this study shows the association between fecal Bacteroidetes levels, propionic acid concentrations, and PVP in patients with liver patients. These findings support the growing evidence implicating the GM and SCFAs in the pathogenesis of portal hypertension (Fig 4c).

## Ethics approval statement and informed consent

This prospective study was approved by the ethics committee of Kyushu University (approval code: 2019‐067). We obtained informed consent from all patients included in study.

## Supporting information


**Data S1.** Supporting Information.

## Data Availability

All data generated or analyzed during this study are included in this article and its supplementary material files. Further enquiries can be directed to the corresponding author.
