# Supplementary material for: Association of gut microbiota with portal vein pressure in patients with liver cirrhosis undergoing living donor liver transplantation
Source: JGH Open. 2023 Dec 9;7(12):982–9. doi: 10.1002/jgh3.13018 (PMC10757484; doi:10.1002/jgh3.13018)
Supplement: Supplementary file 1 — Data S1. Supporting Information. [file JGH3-7-982-s001.docx]

Supplemental Table 1. Detailed clinical characteristics of each recipient

| No. | Age | Sex | Body mass index | Etiology | MELD  score | Child-Pugh  score | ABO incompatible | PVP  (mmHg) |
| --- | --- | --- | --- | --- | --- | --- | --- | --- |
| 1 | 52 | Female | 18.3 | PBC | 17 | 10 | No | 19 |
| 2 | 55 | Female | 20.4 | HCV | 13 | 8 | No | 14 |
| 3 | 39 | Male | 26.5 | Alcoholic | 15 | 10 | No | 15 |
| 4 | 39 | Female | 20.3 | PBC | 23 | 12 | No | 10 |
| 5 | 24 | Male | 21.1 | Wilson disease | 26 | 10 | No | 24 |
| 6 | 68 | Female | 25.7 | HCV | 18 | 12 | No | 20 |
| 7 | 44 | Male | 23.0 | HBV | 12 | 9 | Yes | 20 |
| 8 | 57 | Male | 22.1 | Alcoholic | 10 | 11 | Yes | 22 |
| 9 | 65 | Male | 25.0 | NASH | 12 | 10 | Yes | 14 |
| 10 | 55 | Male | 25.6 | HCV | 19 | 10 | No | 23 |
| 11 | 63 | Male | 21.9 | NASH | 12 | 10 | Yes | 31 |
| 12 | 56 | Female | 26.1 | HBV | 20 | 10 | Yes | 26 |
| 13 | 67 | Male | 23.4 | HCV | 12 | 11 | Yes | 24 |
| 14 | 32 | Male | 20.4 | PSC | 15 | 10 | Yes | 24 |
| 15 | 34 | Female | 17.3 | Alcoholic | 16 | 10 | No | 25 |
| 16 | 62 | Female | 24.0 | HCV | 22 | 9 | No | 23 |

MELD, The Model for End-Stage Liver Disease; PVP, portal vein pressure; PBC, primary biliary cholangitis; HCV, hepatitis C virus; HCV, hepatitis C virus; NASH, non-alcoholic steatohepatitis; PSC, primary sclerosing cholangitis;


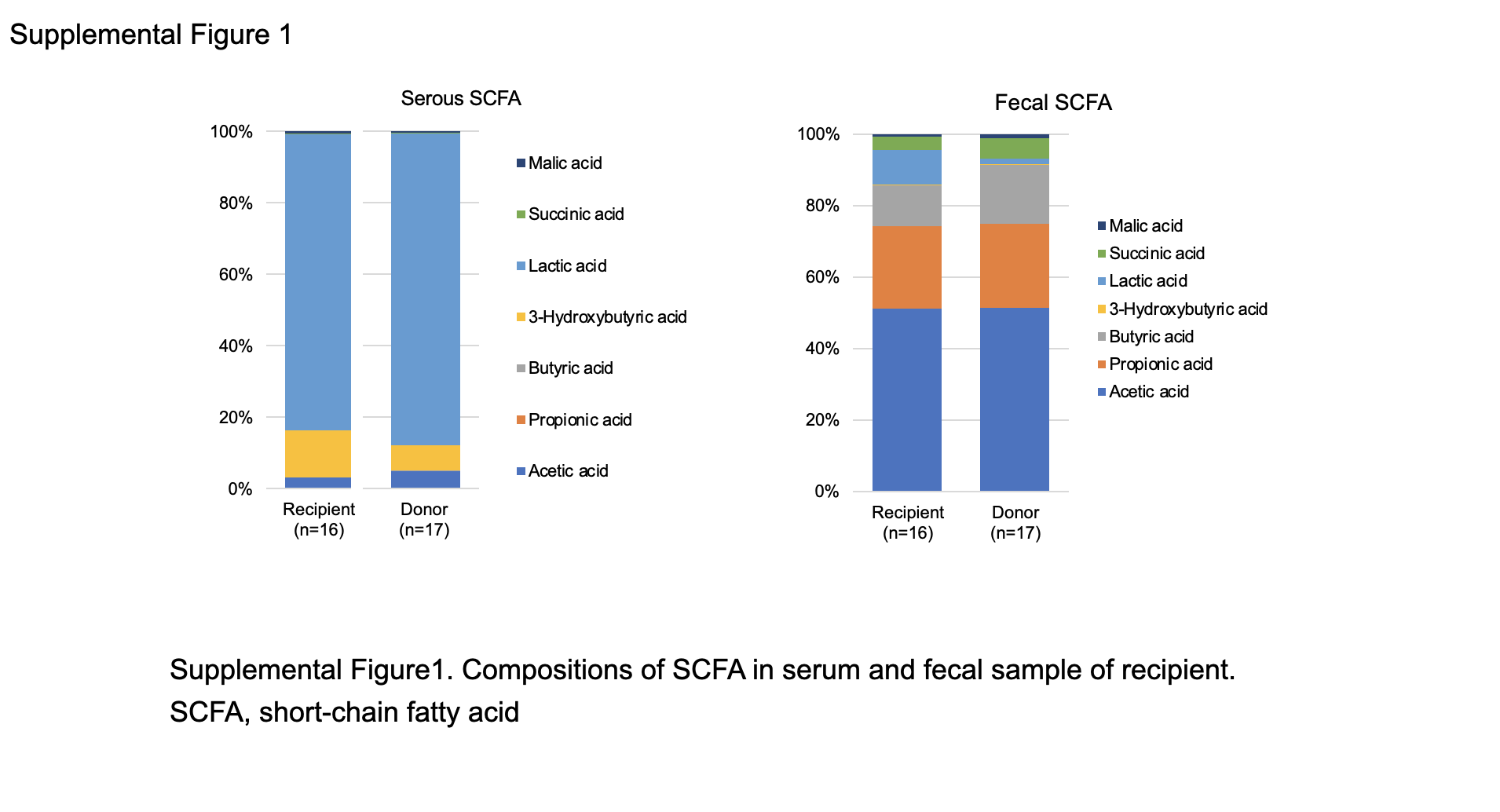


Supplemental Figure1. Compositions of SCFA in serum and fecal sample of recipient.

SCFA, short-chain fatty acid


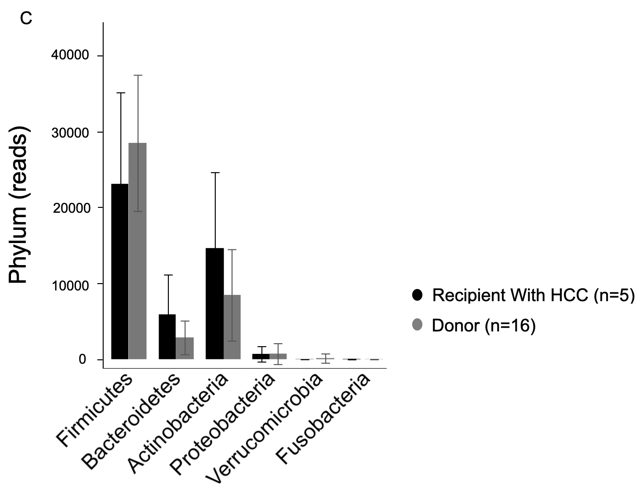

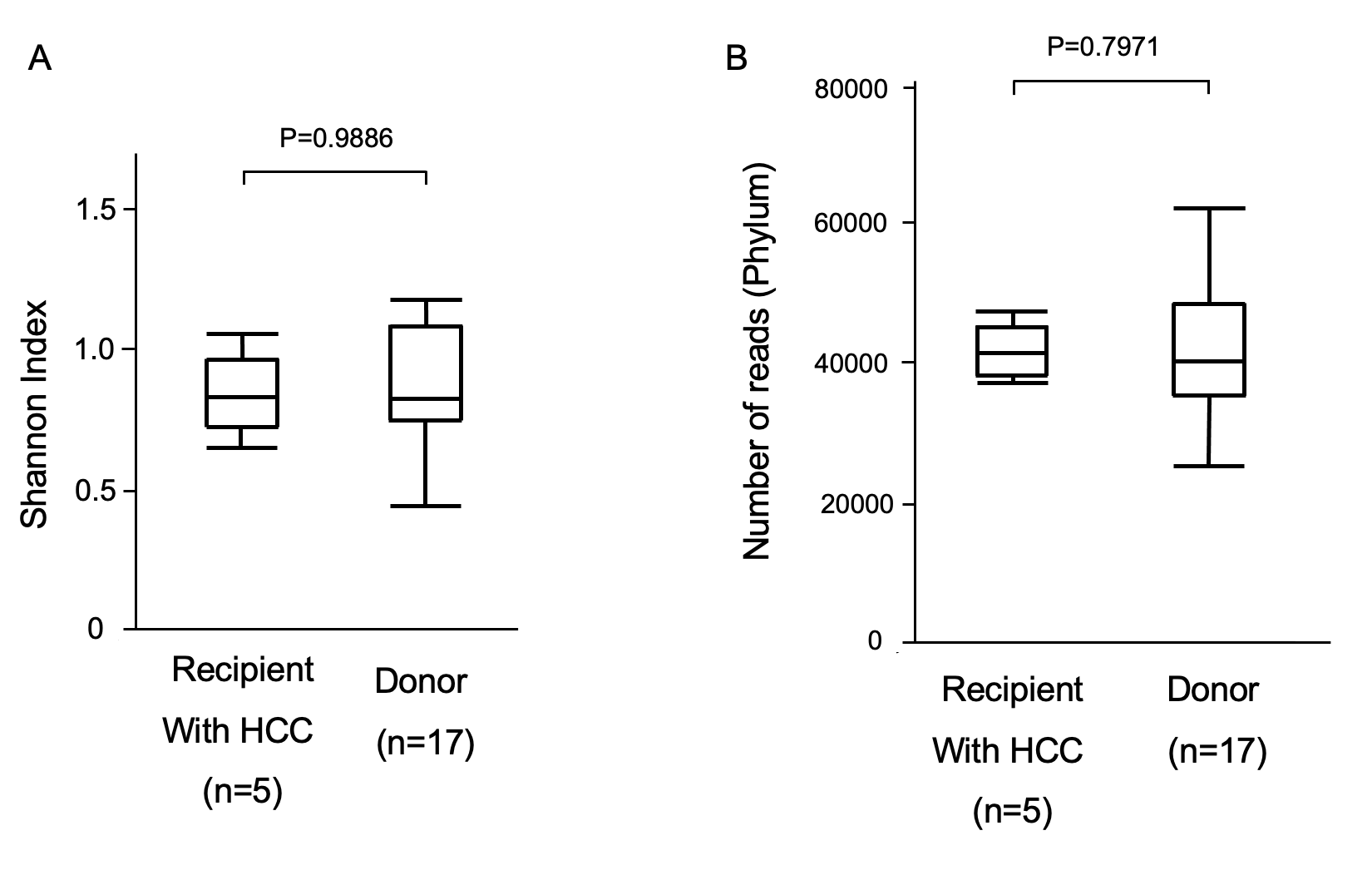


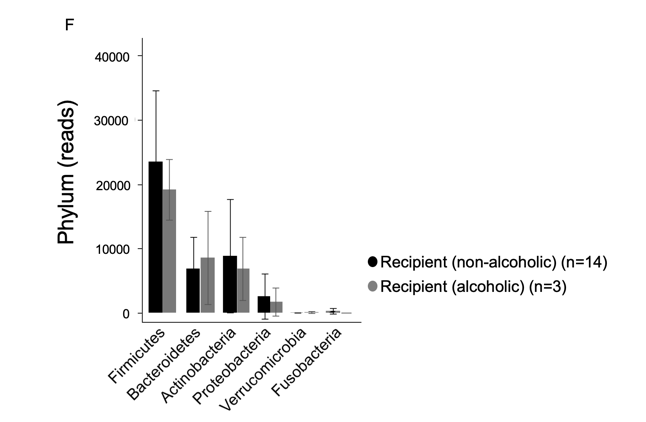

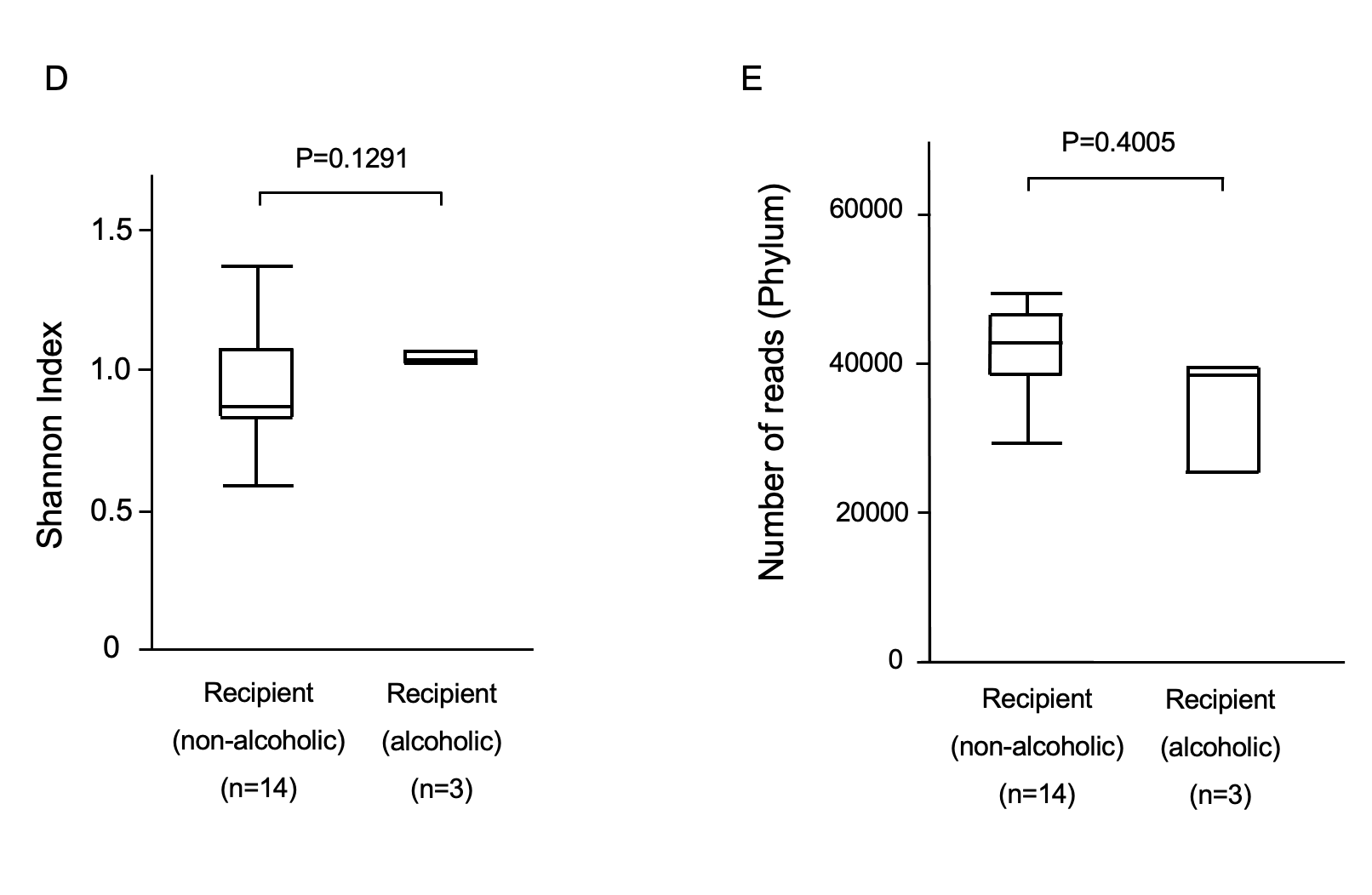


Supplemental Figure 2. Analysis of GM in subgroup

(A) Shannon index of fecal samples between recipients with HCC and healthy donor

1. Leads number of fecal samples (phylum) between recipients with HCC and healthy donor
2. Comparison of GM (Phylum) between recipients with HCC and healthy donor

(D) Shannon index of fecal samples between recipients whose etiology is non-alcoholic or alcoholic

(E) Leads number of fecal samples (phylum) between recipients whose etiology is non-alcoholic or alcoholic

(F) Comparison of GM (Phylum) between recipients whose etiology is non-alcoholic or alcoholic

GM, gut microbiota; HCC, hepatocellular carcinoma


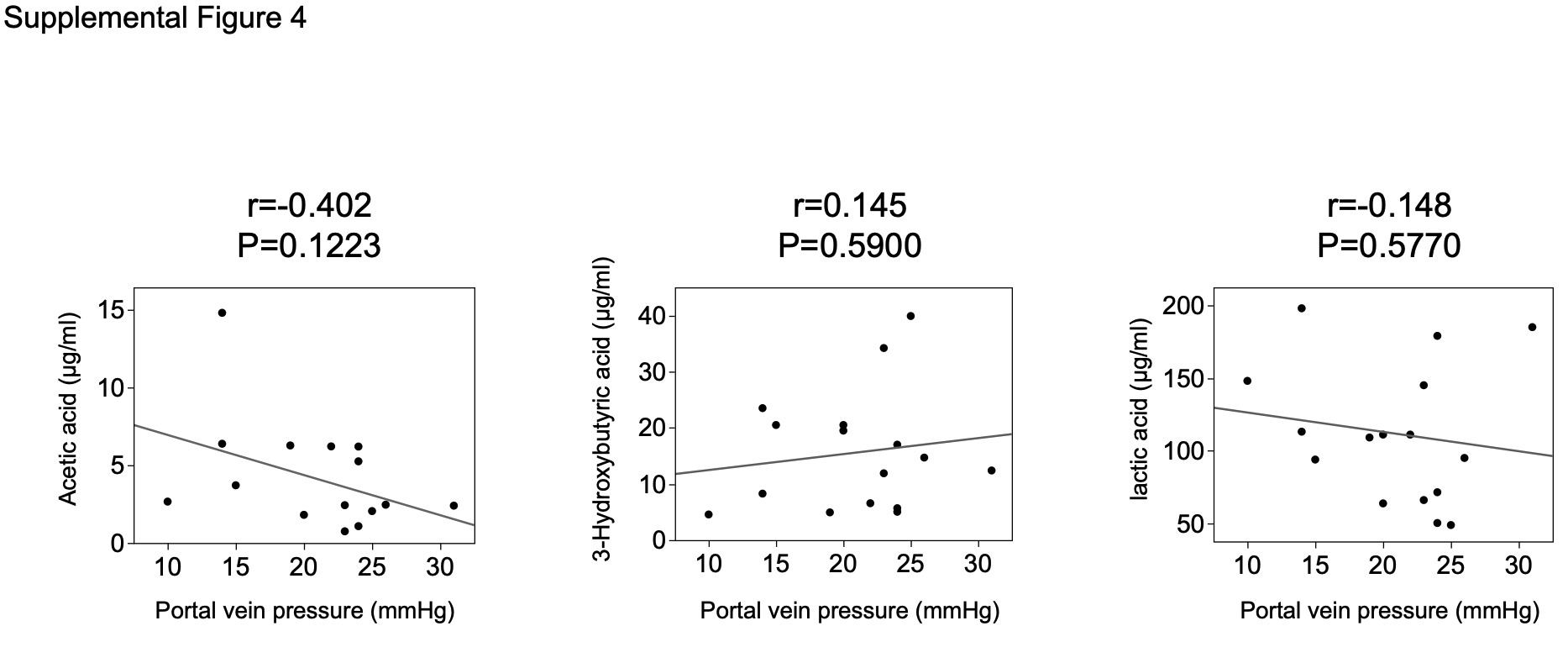


Supplemental Figure 3. Correlation between serum SCFA and PVP

PVP, portal vein pressure; SCFA, short-chain fatty acid


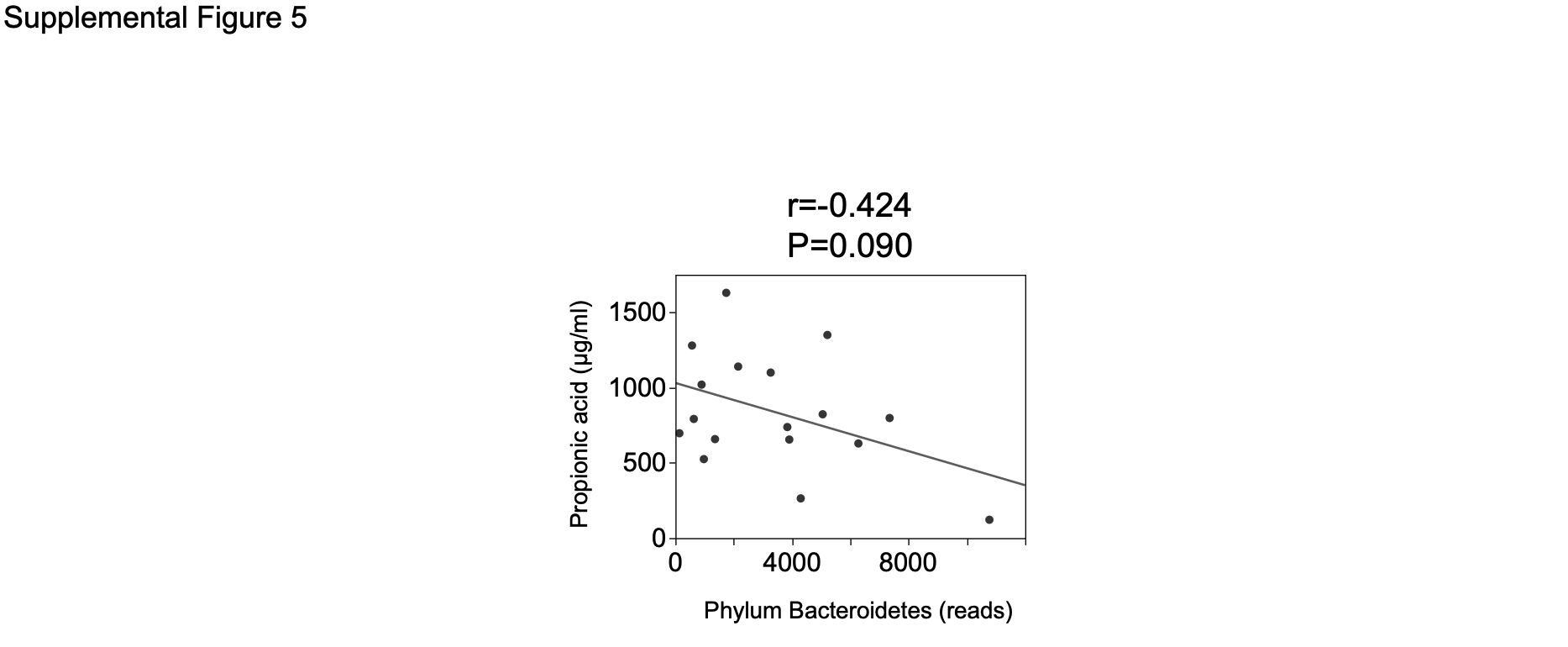


Supplemental Figure 4. Correlation between serum propionic acid and Phylum Bacteroidetes


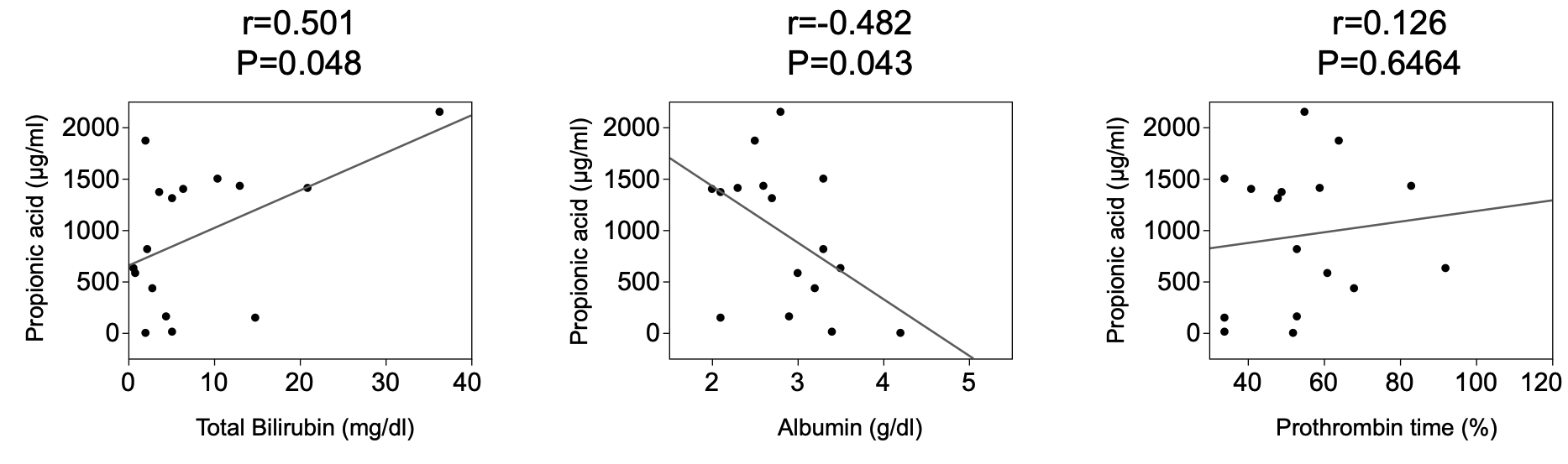


Supplemental Figure 5. Correlation between fecal propionic acid and blood test


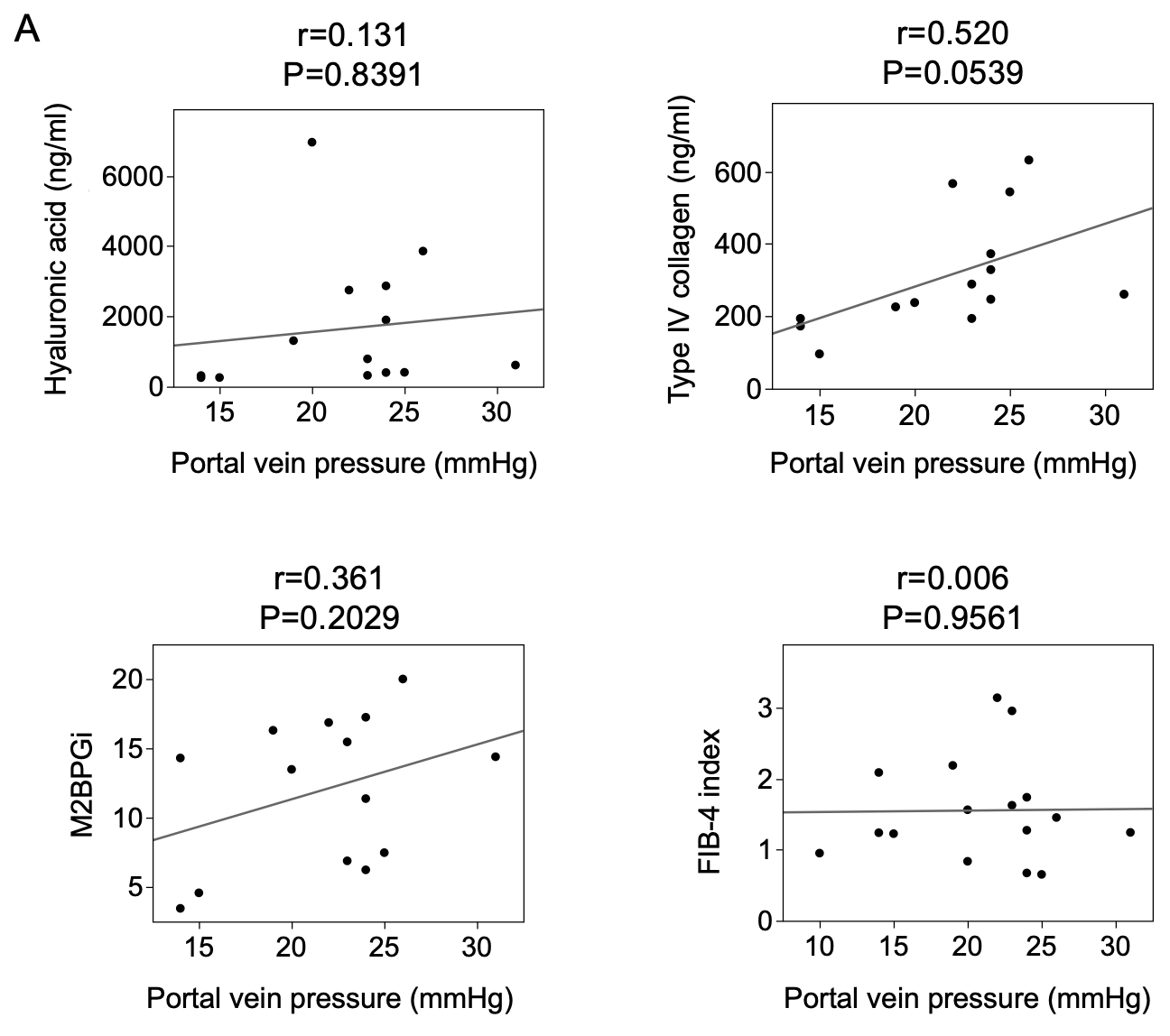


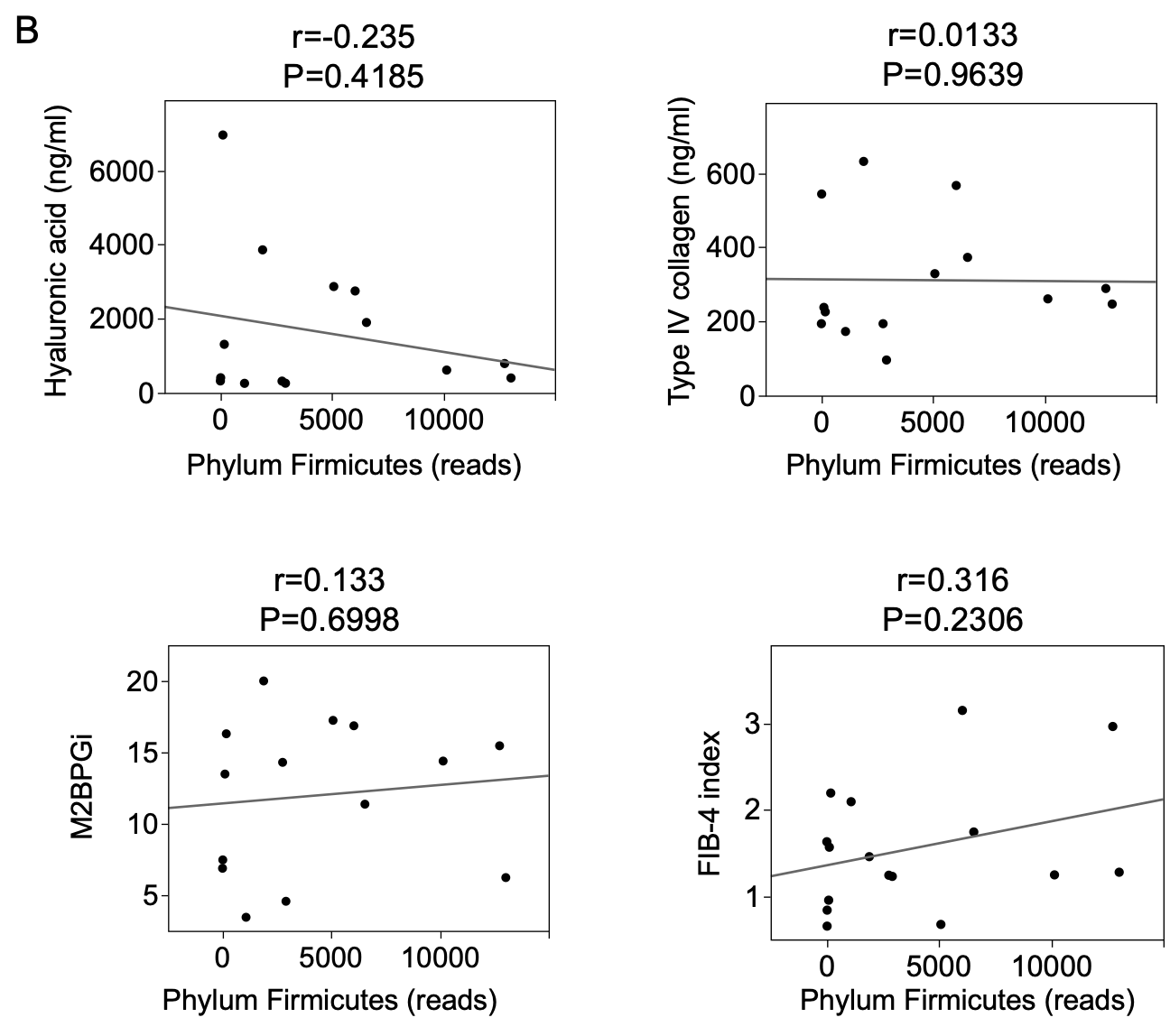


Supplemental Figure 6.

(A) Correlation between liver fibrosis markers (hyaluronic acid, type iv collagen, M2BPGi, FIB-4 index) and PVP

(B) Correlation between liver fibrosis markers (hyaluronic acid, type iv collagen, M2BPGi, FIB-4 index) and phylum Bacteroidetes

FIB-4 index, fibrosis-4 index M2BPGi, Mac-2 binding protein glycosylated isomers; PVP, portal vein pressure
